# Coating *Lacticaseibacillus rhamnosus* GG in Alginate Systems: an Emerging Strategy Towards Improved Viability in Orange Juice

**DOI:** 10.1208/s12249-021-01996-x

**Published:** 2021-04-05

**Authors:** Angela Bonaccorso, Nunziatina Russo, Alessia Romeo, Claudia Carbone, Maria Aurora Grimaudo, Carmen Alvarez-Lorenzo, Cinzia Randazzo, Teresa Musumeci, Cinzia Caggia

**Affiliations:** 1 Department of Drug Sciences and Health, University of Catania, Via Santa Sofia, 64, 6, 95125 Catania, Italy; 2 Department of Agriculture, Food and Environment (Di3A), University of Catania, Via Santa Sofia 100, 95123 Catania, Italy; 3 Departamento de Farmacología, Farmacia y Tecnología Farmacéutica, I+D Farma Group (GI-1645), Facultad de Farmacia and Health Research Institute of Santiago de Compostela (IDIS), Universidade de Santiago de Compostela, 15782 Santiago de Compostela, Spain

**Keywords:** probiotics, lactobacillus encapsulation, experimental design, alginate polymer, functional food

## Abstract

Fruit juices are successfully proposed as suitable probiotic vehicles, but researchers’ efforts should be developed to avoid effects of bacteria overgrowing on sensory and nutritional cues of final products and to preserve viability of probiotic bacteria during storage. In the present study, encapsulation of *Lacticaseibacillus rhamnosus* GG strain in alginate systems was performed through ionotropic gelation technology. The alginate systems were optimized by using Box-Behnken Design to investigate the influence of three independent variables at three different levels: particle mean size and polydispersity index. The optimized probiotic-loaded alginate particles were added to orange juice samples. The viability of the probiotic strain, both as free and microencapsulated, was evaluated in orange juice stored at 5°C for 35 days. Morphology and size of probiotic-loaded alginate particles were found suitable for incorporation into juice. TEM analysis revealed that unloaded systems were clustered as nanoparticles (CL_NP), while the loaded sample appeared as a coated system (Coated_LGG). Microbiological evaluation revealed that the encapsulation assured the survival of Coated_LGG, with a reduction of less than 1-unit log in cellular density after 35 days of refrigerated storage in orange juice. Results indicated that the encapsulated bacteria did not affect the macroscopic properties neither the microbiological characteristic of orange juice; thus, it can be proposed as functional food.

**Supplementary Information:**

The online version contains supplementary material available at 10.1208/s12249-021-01996-x.

## INTRODUCTION

Over the past decade, there was a growing consumer interest in modern food products capable of performing therapeutic and curative features. There is a consistent rise in the demand for functional foods supplemented with probiotics, which are known to improve human health apart from the native nutritional value (1). Probiotics are living microorganisms that, when administered in adequate amounts, confer beneficial effects to the host (2). The health effects of probiotics are strain-specific and dose-dependent (3). Recently, non-dairy products have been considered as an alternative for consumers who are lactose intolerant, allergic to milk proteins, hypercholesterolemic, or strictly vegetarian (3). Consequently, several fermented vegetables or fruit-based products (4,5) are being proposed and forecasted to take a large share of the global market, representing one of the main challenging strategy for food industry (6,7). Fruits and vegetables have been proven to support the survival of probiotics, thanks to their high sugar and total antioxidant content and relatively low pH values. In fruit juices, probiotic cultures could be added as biomass (8,9) or through direct addition of lyophilized cultures (10). However, these methods besides being time consuming, requiring specific equipment (as refrigerated centrifuges) and specialized operators (8), can reduce the microbial viability and impair sensory properties (11–13). Moreover, it is well-known that to obtain a temporary colonization of the intestine “the minimum density of required probiotic is at least 1 billion live cells per strain per day” (3,6,14). In order to overcome the aforementioned disadvantages and to guarantee such a high concentration, microencapsulation can be exploited as a promising strategy (11,15).

Microcapsules (MCs) consist of a core containing active ingredients and a polymeric shell, acting as a protective membrane able to regulate substances exchange in and out (15). Polymers of natural origin are mostly used for probiotic microencapsulation (12,13); among them, alginate is the most extensively used to microencapsulate lactobacilli and bifidobacteria (16). The choice of the adequate preparation method of MCs depends on several factors such as physicochemical properties of the selected materials, desired particle size, and release pattern. Among the described methodologies, the ionotropic gelation technique allows to obtain microparticles under mild conditions; as an example, an alginate solution is usually dripped into a cross-linking solution consisting of Ba^+^, Ca^2+^, and Sr^2+^ salts (16). When the bivalent cation comes into contact to alginate chains, interchain bonds are established generating a three-dimensional polymeric network, defined as “egg-box” like for the characteristic shape of the supramolecular structure (17).

The aim of the present study was to design alginate-based particles suitable for loading *Lacticaseibacillus rhamnosus* GG cells into fruit juices with multivariate approach of quality by design using Box-Behnken Design (BBD) (18,19). The critical quality attributes (CQAs) and the critical process parameters (CPPs) were selected from the knowledge space through literature analysis of the last 10 years (PubMed sources) (20–22). In order to optimize the formulation, the quality target profile (QTTP) was defined. The effect of three independent variables was investigated at three different levels on particle mean size and polydispersity index (PDI). The optimized alginate system was loaded with *Lacticaseibacillus rhamnosus* GG (23,24), and their behavior was investigated in low-pH juices. Moreover, the viability of the probiotic strain, both as free and microencapsulated, was evaluated in orange juice stored at 5°C for 35 days.

## MATERIALS AND METHODS

### Materials

Sodium alginate (20–40 kDa), sunflower oil from *Helianthus annuus*, and Span 80 were purchased from Aldrich S.r.l. (Milan, Italy). Calcium chloride was obtained from Riedel-de-Haën (Hannover, Germany). Deionized water was used for all preparations. Sicilian 100% blood orange juice (pH 3.05), with no added sugar o preservatives, was kindly provided by the Oranfrizer Company (Scordia, Sicily).

### Preparation of Probiotic Culture Cells

*L. rhamnosus* GG (LGG), belonging to the Department of Agriculture, Food and Environment (Di3A, University of Catania) was cultured in MRS broth medium (Liofilchem, Italy) and anaerobically incubated at 37°C for 18 h. The cells were harvested by centrifugation (BagMixer, Interscience, France) at 4500×*g* for 10 min at 4°C. The pellets were washed twice with phosphate-buffered saline (PBS, pH 6.8) and collected by centrifugation as above. The obtained washed pellets, at a final cell concentration of approximately of 10^10^ colony-forming unit/mL (CFU/mL), were refrigerated and immediately transferred to the Laboratory of Drug Delivery Technology for further treatments.

### Design of Experiment (DoE)

The optimization of formulation variables was carried out using BBD. The experimental design was generated using the Design-Expert software (7.0.0, Stat-Ease Inc., Minneapolis, MN). Response surface quadratic model was performed for the optimization of formulation variables and the evaluation of the optimum level of each factor.

Three independent variables at three coded levels were selected (Table I): span 80 concentration (*X*_1_), alginate concentration (*X*_2_), and the aqueous to oil phase (A/O) ratio (*X*_3_). The coded variables *X*_1_, *X*_2_, and *X*_3_ correspond to *A*, *B*, and *C*, respectively, within the equations provided by the software. In accordance with the design, 17 formulations were prepared, and the effect of each factor was studied on the particle size (*Y*_1_) and PDI (*Y*_2_) of the formulation as response variables. The experiments were randomized to avoid experimental bias. Three-dimensional (3D) response surface graphs were generated for diagrammatic depiction of values of response. Statistical analysis was performed by using the ANOVA software.

**Table I:** Factors and the Corresponding Levels Investigated During the Box-Behnken Design

Coded Factors	Coded levels	
	Low	High
*X*_1_: span conc (% w/v)	0.1	0.5
*X*_2_: alginate conc (% w/v)	0.5	2
*X*_3:_ A/O (v/v)	1:20	1:6

### Alginate-Based System Optimization

Formulation optimization was performed using the “desirability tool,” provided by the Design-Expert® (7.0.0, Stat-Ease Inc., Minneapolis, MN) software. This is a method for simultaneous optimization of dependent variables, which permits the calculation of an objective function, and results are obtained with values ranging from 0 (undesired response) to 1 (a fully desired response). The levels of all independent variables are then automatically combined to identify the optimal conditions within the experimental domain. The desirability for a given combination of variables is calculated as the geometric mean of all individual desirability for each response.

### Preparation of Alginate-Based System by Ionotropic Gelation

The alginate-based particles were prepared by the ionotropic gelation method. Briefly, sodium alginate (Table I, from 0.5 to 2.0% w/v) was dissolved in deionized water. The aqueous phase was added dropwise to a sunflower seed oily phase containing different concentration of Span 80 (Table I), under magnetic stirring (1000 rpm). The two phases were mixed in different ratios, according to the experimental design, and then maintained on a magnetic plate under constant stirring. To obtain a double water-in-oil-in-water emulsion (W/O/W), a given volume of the above-described emulsion was added under constant stirring dropwise to the same volume of calcium chloride solution (0.45 M). At the end of the procedure, the double emulsion was maintained for 30 min at room temperature. The obtained alginate-based particles were purified through centrifugation (5000 rpm for 30 min at 6°C), using a Thermo Scientific SL16R centrifuge (Thermo Fisher Scientific Inc., MA, USA). The supernatant was discarded, and the particles collected with deionized water on filter paper and washed with water. Finally, particles were weighed and re-suspended in 10 mL of deionized water and analyzed using photon correlation spectroscopy (PCS).

### Particle Size Distribution and Zeta Potential Measurements of Alginate-Based Systems

PCS was applied to evaluate mean size, PDI, and ZP of alginate-based particles in suspension, using a Zetasizer Nano S90 (Malvern Instrument, Malvern, UK). The cuvette containing the dispersions was illuminated by a 4 mW He-Na laser beam with a light source (633-nm wavelength). The ZP values were determined at 25°C. All measurements were performed in triplicate, and the results expressed as mean ± standard deviation (SD).

### Preparation of Optimized Alginate-Based Particles Loaded with *Lacticaseibacillus rhamnosus* GG Cells

The optimized formulation (CL_NP) suggested by BBD was composed as follows: *X*_1_ 0.42% w/v; *X*_2_ 2% w/v; *X*_3_ 1:20 v/v. The probiotic-loaded alginate-based particles were prepared using the same procedure, wherein the bacterial cells (9.11 log_10_ CFU/mL) were added in lyophilized form to the sodium alginate aqueous phase (probiotic final concentration 1% w/v) before the preparation of the primary emulsion. The obtained Coated_LGG particles were freeze dried using an Edwards Modulyo freeze dryer (Thermo, Waltham, MA, USA) for 24 h at 2 mbar to produce the dry powder. The resultant lyophilized Coated_LGG particles were resuspended in distilled water or Sicilian 100% blood orange juice (pH 3.05), to characterize the optimized probiotic-loaded alginate-based particles or for stability and viability tests, respectively.

### Transmission Electron Microscopy

The *Lacticaseibacillus rhamnosus* GG cells, empty optimized alginate-based particles (CL_NP), and optimized alginate-based particles (Coated_LGG) were subjected to morphological analysis. The transmission electron microscopy (TEM) analysis was performed using a high-resolution microscope JEM-1011 (JEOL USA Inc., Peabody, MA, USA). The pellet of alginate-based particles was resuspended into 10 mL of distilled water, and an aliquot was placed on carbon-coated grids. After the addition of few drops of phosphotungstic acid, the samples were dried before observation.

### Stability Evaluation

The stability of empty (CL_NP) and loaded particles (Coated_LGG) was evaluated resuspending the pellets into orange juice. The fruit juice without any addition was used as control (CTRL).

Each sample was subjected to mean size, PDI, ZP (Zetasizer Nano S90, Malvern Instrument, Malvern, UK), and pH evaluation (pH-meter Mettler Toledo, Columbus, OH, USA) after 5 h of storage at 25 and 5°C (T0) and after 1 week of storage at the same temperatures. Measurements were performed in triplicate and results expressed as the mean ± SD.

Stability was also monitored for 1 week through Turbiscan® Ageing Station (TAGS, Formulaction, L’Union, France) by placing each sample (20 mL) in a cylindrical glass cell positioned in the Turbiscan® at 25°C. The detection head was composed of a pulsed near-infrared light source (880 nm) and two synchronous transmission (T) and backscattering (BS) detectors. The T detector received the light, which crossed the sample (at 180° from the incident beam). The detection head scanned the entire height of the sample cell (65-mm longitude), acquiring T each 40 μm (1625 acquisitions in each scan).

### Counting of Viable Encapsulated and Free LGG Cells

In order to count the encapsulated LGG viable cells both in loaded alginate-based system and in juice samples added with Coated_LGG, a treatment with phosphate buffer (PBS, pH 6.8) was applied. In details, 1 g of Coated_LGG or 1 mL of juice sample was added to 9 mL of PBS solution and homogenized by gentle shaking at room temperature for 10 min (until complete rupture), using an orbital shaker (IKA KS 250 basic, Labortechnik), in order to induce the alginate shell disruption and to liberate the microorganisms. The released viable LGG cells were then counted by spread plating method in MRS agar anaerobically incubated at 37 °C for 48 h and expressed as log_10_ colony-forming unit per gram-Ml (CFU/g-mL). The counting of free or failed coated lactobacilli cells was carried out by serial dilution in saline solution (0.9% w/v of NaCl) and plating on MRS agar and expressed as above.

### Physicochemical and Microbiological Analyses of Orange Juice Samples

In the present study, a Sicilian 100% blood orange juice (pH 3.05, without any addition of sugar or preservatives) was kindly provided by the Oranfrizer Company (located in Scordia, Sicily) and used as probiotic delivery matrix. Empty CL_NP, Coated_LGG, and LGG free cells were aseptically added into 100 mL of orange juice. Samples were gently mixed, stored, and subjected to physicochemical and microbiological analyses. The pH value of samples was measured at 0, 7, 14, and 35 days of storage, as described above. Total soluble solid (TSS) value, as °Brix, was determined using a refractometer (Atago, RX-5000).

Samples from all treatments were analyzed in triplicate for viable count, and results expressed as mean log CFU/mL ± SD. Comparison between free and encapsulated cell survivability was carried out. In addition, in order to evaluate the microbiological quality of juice for each sample, the viable count of *Leuconostoc*, mesophilic aerobic bacteria, psychrotrophic bacteria and yeasts/molds were detected during storage time, using Mayeux, Sandine, and Elliker (MSE), Plate Count Agar (PCA), and Sabouraud Dextrose Agar (SDA) media, incubated at 32, 15, and 25°C, respectively.

### Statistical Analysis

All experiments were carried out in triplicate, and results presented as mean ± SD. A single-factor ANOVA was used to compare the treatments. Significant differences between the means of cell counts were determined using Tukey’s HSD test. All statistical analyses were carried out using Minitab v17.3.0 (Minitab Statistical Software. Minitab Inc., USA). Results were considered statistically significant when *p* < 0.05.

For the statistical analysis of CL_NP and Coated_LGG (mean size and PDI) during stability study, *t*-test was performed using Prism 6 (GraphPad Software, Inc., La Jolla, CA, USA). Significance was defined as *p* < 0.05.

## RESULTS AND DISCUSSION

### Preparation of Alginate Microcapsules

In the present study, alginate-based particles were successfully prepared by ionotropic gelation method. The design of the formulation was performed after a preliminary investigation necessary to tune the particle preparation procedure. In particular, different factors that can affect the microencapsulation procedure, also known as “CPPs” were taken into account. The process parameters at the lowest and highest level investigated are summarized in Supplementary Table 2. The selection of specific parameter was performed based on preliminary tests and on the results reported in literature. This step was very crucial to maintain each parameter at constant value during the preparation method. Accordingly, magnetic stirring was kept at 1000 rpm; the dripping distance did not affect the procedure; thus, this parameter was not considered; particle purification was performed at 5000 rpm at 6°C for 30 min. Finally, the crosslinking time was fixed at 30 min.

In order to minimize the experimental runs, the optimization of microencapsulation process was performed applying the BBD, based on three coded levels of the three independent variables (Table I).

The independent variables (Span® 80 concentration, alginate concentration, and W/O ratio) were set based on preliminary screening. As the concentration of surfactant (*X*_1_) affects the stability of the W/O emulsion, the choice should be limited to surfactants with a low HLB value (in a range between 3 and 6). For this reason we selected Span® 80 (HLB = 4.3), and its concentration was evaluated in the range 0.1–0.5% w/v, according to previous observations (24). Alginate concentration affected particles size, and an inverse correlation between diameter and polymer concentration (*X*_2_) was observed. In agreement with other reports, small particle size was obtained using 2% w/v alginate (25). Furthermore, since an increase in alginate concentration increases the viscosity, which could hinder syringing during the dripping, in the present study, alginate concentrations tested were set as 0.5 and 2.0% w/v as low and high values, respectively (22,25). As already reported, additional variables, such as CaCl_2_ concentration and mixing speed, can affect particle properties during microencapsulation process. In the present work, these parameters were kept constant at 0.45 M and 1000 rpm, respectively. The CaCl_2_ concentration affects the integrity of MCs outer membrane and should assure an optimal coverage (22,25). In fact, the droplets obtained during the preparation procedure gelled on surface thanks to the ionic complex formation between alginate and calcium ions, whereas particle diameter was found to be inversely related to the mixing speed (26).

### Effect of Independent Variables on Particles Size and PDI

Seventeen formulations were prepared, and their properties are shown in Table II.

**Table II:** Formulations Prepared According to BBD

	Independent variables^a^			Dependent variables^b^	
Run	*X*_1_	*X*_2_	*X*_3_	*Y*_1_	*Y*_2_
1	0.5	1.25	1:20	657.5 ± 129.0	0.228 ± 0.086
2	0.5	1.25	1:6	750.0 ±21.1	0.375 ± 0.323
3	0.3	0.5	1:20	918.0 ± 9345	0.678 ± 0,558
4	0.1	2	1:10	1372.0 ± 163.7	0.194 ± 0.143
5	0.3	2	1:6	1057.0 ± 137.8	0.436 ± 0.342
6	0.3	1.25	1:10	913.8 ± 20.6	0.287 ± 0.237
7	0.3	0.5	1:6	986.0 ± 607.3	0.696 ± 0.275
8	0.1	1.25	1:20	1317.0 ± 124.5	0.699 ± 0.254
9	0.3	1.25	1:10	924.0 ± 260.2	0.273 ± 0.142
10	0.5	0.5	1:10	903.5 ± 98.72	0.776 ± 0.168
11	0.1	1.25	1:6	1649.1 ± 109.9	0.520 ± 0.425
12	0.3	1.25	1:10	920.0 ± 1124	0.232 ± 0.118
13	0.5	2	1:10	644.5 ± 37.3	0.410 ± 0.218
14	0.1	0.5	1:10	1925 ± 906.5	1
15	0.3	1.25	1:10	978.2 ± 222.2	0.257 ± 0.164
16	0.3	1.25	1:10	976.0 ± 290.1	0.149 ± 0.107
17	0.3	2	1:20	709.0 ± 21.83	0.527 ± 0.112

^*a*^*Independent variables: X*_*1*_ *= [Span 80]; X*_*2*_ *= [alginate]; X*_*3*_ *= water:oil*

^*b*^*Dependent variables: Y*_*1*_ *= size (nm) ± SD; Y*_*2*_ *= PDI± SD*

Particles size and PDI were firstly correlated with the adopted independent variables through the full quadratic equations. The ANOVA analysis of the full regression models showed that only some factors (linear, quadratic, or interaction) were statistically significant; thus, statistically non-significant terms were removed with the exception of terms needed for hierarchy in the reduced regression model. The second-degree polynomial equations, describing the mathematical relationships between the independent and response variables obtained in the reduced regression models, are shown in Eqs. (1)–(2):

1 $$ Size=+965.02-225.84\ast A+1.36\ast B+133.15\ast {B}^2 $$

2 $$ PDI=+0.25\hbox{--} 0.028\ast A-0.24\ast B+0.074\ast C+0.11\ast A\ast B+0.10\ast {A}^2+0.26\ast {B}^2 $$

The quality of the fit of the experimental data using the reduced quadratic models was assessed based on several statistical criteria (Table III).

**Table III:** Analysis of Variance Results

	Particle size			Polydispersity		
Polinomyal term	Coeff. estimate		*P* value	Coeff. estimate		*P* value
Model		Quadratic^a^	0.0002		Quadratic^a^	<0.0001
*A*	−225.84		<0.0001	−0.028		0.3222
*B*	1.36		0.9722	−0.24		<0.0001
*C*				0.074		0.0199
AB				0.11		0.0160
AC						
BC						
*A*^2^				0.10		0.0206
*B*^2^	133.15		0.0247	0.26		<0.0001
*C*^2^						
*R*^2^		0.7615			0.9380	
*F* value		13.83			25.19	

^*a*^*Reduced quadratic model*

As reported in Table III, the *F*-value, the *p*-value, and the *R*^2^ revealed that the chosen quadratic models were significant, demonstrating that the quadratic model was adequate and satisfactorily explained the data for both size and PDI responses. The value of the model’s adequate precision was much higher than the critical value of 4 (11.201 for *Y*_1_ and 16.081 for *Y*_2_ respectively), showing that the model was adequate for predicting particle size and PDI in the applied experimental domain.

The estimated coefficients (Table III) represented the contribution of each individual variable on the response (27), with the positive and negative signs indicating a synergistic or a negative relationship between variables (28).

In the case of particle size, *A* and *B*^2^ were the most significant model terms. During stepwise regression procedure, insignificant terms were removed from the final quadratic model equation, with the exception of the linear coefficient of *B*, which was kept to maintain the model’s hierarchy. The significance of the squared term determines whether there is a quadratic effect. In fact, in our case, *B* is a significant term but its influence on *Y*_1_ can be represented as a curvilinear relationship.

**Fig. 1:**
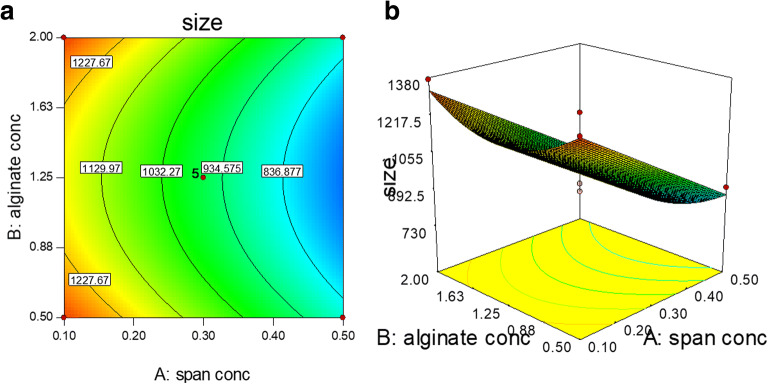
Contour plot (**a**) and 3D surface (**b**) of the effect of alginate (mg/ml) versus Span80 concentration (%) on the particles size

As reported in Fig. 1 (a, b), the significant quadratic term (*B*^2^) indicates that the response decreases in the middle of the design space (valley in the surface) compared to the corners of the design space.

The coefficient estimated for *A* was negative showing that along with the increase of the surfactant concentration, the mean particle size decreased (29). By reducing the surface tension, smaller droplets were formed; during the nucleation phase, a faster removal of particles from the emulsion occurred, facilitating the formation of particles with reduced dimensions (18,30). Indeed, during the preparation, surfactant covers the developed particles preventing their further growth; furthermore, their surface energy decreased preventing the agglomeration phenomena. The coefficient estimated for *B*^2^ indicated that as the polymer concentration increased, particle diameter gradually increased, as previously reported (25,31).

Furthermore, as shown in Supplementary Figure 1, a good correlation between the actual and predicted values was observed, confirming a good fitting and that the BBD model can be effectively applied for optimization.

**Fig. 2:**
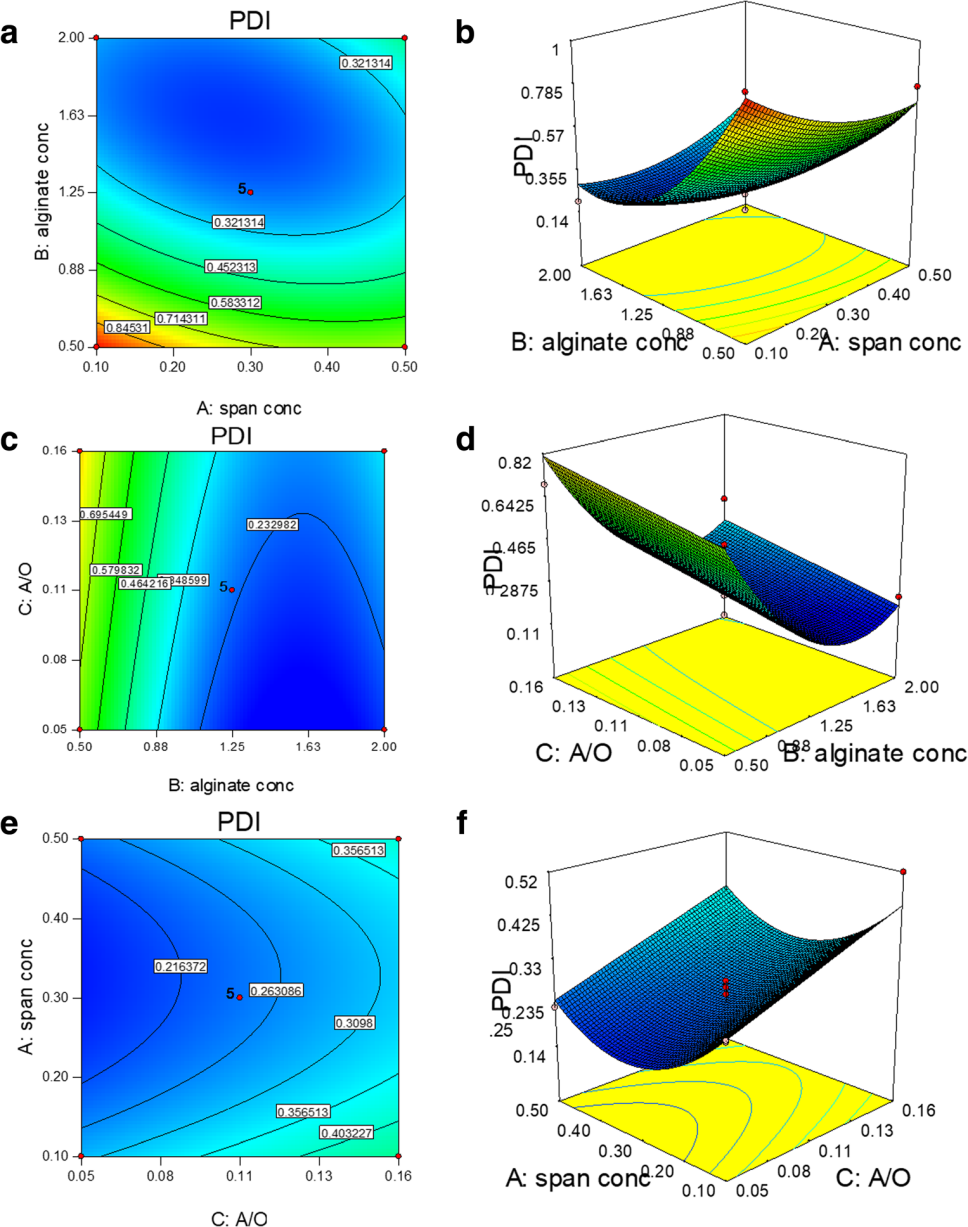
Contour plot (**a**) and 3D surface (**b**) of the effect of alginate (mg/ml) versus Span80 concentration (%); contour plot (**c**) and 3D surface (**d**) of the effect of alginate concentration (mg/ml) versus A/O ratio (v/v); contour plot (**e**) and 3D surface (**f**) of the effect of Span80 concentration (%) versus A/O ratio (v/v) on particles PDI

Regarding PDI response, *B*, *C*, AB, *A*^2^, and *B*^2^ were found to be significant model terms (Fig. 2). All factors affected this response, as individual term and/or in combination. When an interaction term is found to be significant, the correspective linear terms are critical to the model because they are the parent terms to the interaction. The interaction coefficient is basically a correction to the individual parent term coefficient when the second parent is set at its different levels. The interaction that mainly affected the PDI response (*Y*_2_) was AB, which referred to the interaction between Span 80 and alginate concentration.

The interaction contour and 3D surface plots (alginate concentration versus Span concentration) that resulted from the developed reduced quadratic model (Fig. 2a, b) depicted the increase in the PDI with a reduction in both span and alginate concentration, so that a maximum PDI value of 1 is reached at the minimum values of these two factors in the selected experimental domain.

Consequently, high concentrations of surfactant and polymer should be used to obtain homogeneous particles. The surfactant played an important role in stabilizing the particles in suspension. Furthermore, the stability was strictly related to low PDI values. High polydispersity may cause serious drawbacks such as unpredictable behavior (32). Particle surface coverage increases higher surfactant concentration, which may also guarantee the steric resistance of the shell. Conversely, an insufficient surfactant concentration could reduce the system’s stability (33).

### Optimized Alginate System

Once the factors and variables exerting a significant impact on the dependent variables of alginate-based particles were assessed, the formulation candidate to load probiotic cells was optimized. For the numerical optimization, the desired goals for each response (none, minimize, maximize, target, or range) were applied (Supplementary Table I). As showed in Supplementary Table I, the candidate formulation target parameters were particle size near 1000 nm that allowed coating the bacteria cells as a layer. At the same time, this particle diameter maintains the sensorial traits of juice. Then, we set to minimize the PDI to obtain a homogeneous suspension.

In order to determine the best combination of responses, the objective function involved the use of a geometric mean for the search of the greatest overall desirability for responses and/or factors. Desirability values equal to 1 indicated that all goals were satisfied. Differently, values equal to 0 indicated that one or more responses fall outside the acceptable limits.

The formulation proposed with the highest desirability (0.973) was selected and experimentally validated. Its composition was *X*_1_ 0.42% w/v, *X*_2_ 2% w/v, and *X*_3_ 1:20 v/v.

The predicted and observed values were compared, and the error percentage was calculated for each response, as shown in Table IV. All percentage errors were considered satisfactory (<10%). Overall, results showed a good correlation between the observed and predicted values, which confirmed the reliability of the model. The optimized particles showed negative surface charge (−48.6 ± 1.10 mV) due to the presence of alginate.

**Table IV:** Results of Dependent Variables Obtained from the Optimized Formulation: Predicted, Observed Values, and Error Percentage

	Predicted value	Observed value^a^	Error %^b^
*Y*_1_ (nm)	994.56	1047 ± 18.07	5.27
*Y*_2_	0.251	0.266 ± 0.027	5.97

^*a*^*Particle mean size± SD; PDI ± SD*

^*b*^Absolute predicted error = |(Obs.value-Pred.value)/Pred.value|*100

**Fig. 3:**
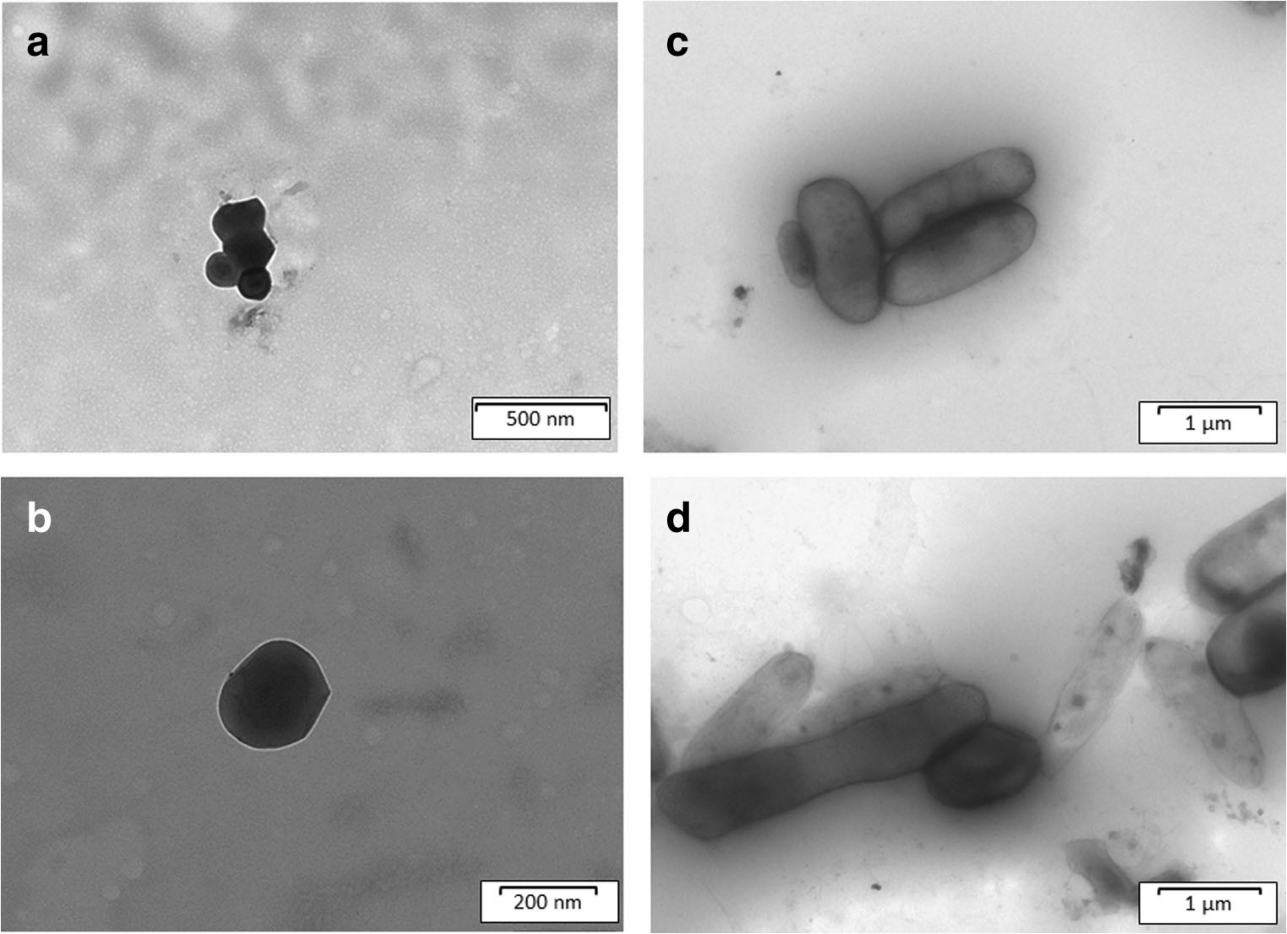
TEM images of optimized empty cluster of nanoparticles (CL_NP) (**a**, **b**); TEM images of coated *Lacticaseibacillus rhamnosus* GG (Coated_LGG) (**c**, **d**)

TEM images of empty optimized formulation are shown in Fig. 3 (a, b). As suggested by PDI values, the images revealed that the optimized CL_NP consists of a cluster of nanoparticles with spherical shape and smooth surface. Moreover, the CL_NP displays a continuous wall free of cracks and surface indentation, essential to provide good protection to the core and prevent an uncontrolled cargo release (34). These observations confirmed that the obtained formulation consisted of capsules, as demonstrated by the different color intensity that well differentiates the shell in the outer part of the particle from the core, located in the innermost zone with a more marked color.

### *Lacticaseibacillus rhamnosus* GG Cell-Loaded Alginate Particles

The optimized formulation was selected for probiotic loading, and a comparison was made between the empty and the loaded systems. When probiotic cells were encapsulated (Coated_LGG), the average size of the systems slightly increased (from 1047 nm to 1165 nm), and TEM analysis revealed that the system modified its structure from a spherical CL_NP (Fig. 3b) as cluster (Fig. 3c) to a structure elongated and with elliptical shape (Fig. 3c, d).

The PDI of loaded formulation decreased from 0.266 to 0.181, indicating a higher particle homogeneity compared to empty CL_NP. The ZP remained negative with values equal to −36 ± 0.557.

In general, the probiotic microencapsulation strategy may allow to obtain particles that consist of one or several cells. When several cells are enclosed by the capsule, the interstitial liquid from solution fills the free spaces of the “multicells microcapsule”. Our results suggest the presence of a single-cell encapsulation with the bacterial cell forming the core of the capsule, surrounded by the polymeric shell. This hypothesis is supported by previous studies by Mortazavian *et al.*, who described the possibility to add an additional second layer to the polymeric capsule, also known as shell, coat, or support layer (35). TEM images of loaded formulation (Fig. 3c, d) highlighted the typical cell morphology of LGG and surrounded by a layer of polymer; in fact, the images did not show other structure that may hypothesize that alginate formed separate nanosystem. During encapsulation, the polymer “adapts” itself to the present cells stratifying along their surface as a protective coating. In this way, we successfully obtained a microcaspular system containing probiotic cell, to preserve the juice properties that will not be affected by the presence of macroscopic visible particles.

### Stability Evaluation

Stability tests were performed during storage of the CL_NP and Coated_LGG formulations in orange juice for 1 week. Samples were kept at 5 and 25°C and subjected to PCS and pH evaluation, while Turbiscan® analysis was performed only at 25°C.

**Fig. 4:**
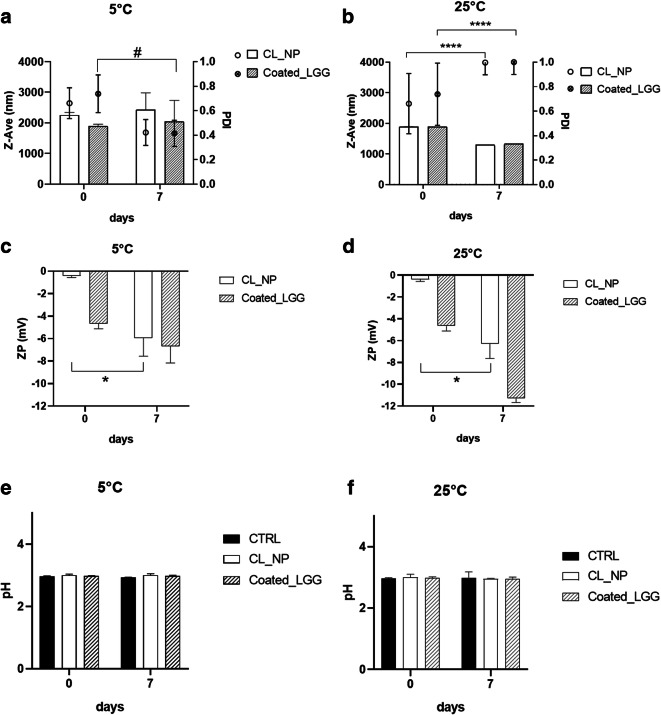
Stability studies: mean size, PDI (**a**), ZP (**c**), and pH (**d**) of the samples stored at 5°C and mean size, PDI (**b**), ZP (**d**), pH (**f**) of the samples stored at 25°C. The asterisk symbol denotes statistical significance difference for size and zeta potential. The number sign symbol denotes statistical significance difference for PDI. Significance was defined as **** *p* < 0.0001

As reported in Fig. 4a, both empty CL_NP and Coated_LGG showed high stability at 5°C; no variation in particle diameter was highlighted, with reduction of PDI values in both formulations. Both samples stored at room temperature showed a reduction of particle diameter and an increase of PDI values (Fig. 4b).

XParticle ZP was affected by the matrix, as revealed by the shift toward neutral values, at 0 time for CL_NP (Fig. 4c, d). The difference in ZP values observed in fruit juice and aqueous suspension is due to the particles’ environment, as the ZP is defined as difference of potential between the dispersion medium and the stationary layer of fluid attached to the dispersed particles. One of the most important factors that affects ZP is the pH of the medium. Even in the case of Coated_LGG, a decrease in the ZP values was observed, which was found to be higher at 25°C. The pH of CL_NP and Coated_LGG was stable along the storage time, as reported in Fig. 4e, f. After 7 days, a reduction of ZP with negative values ~−6mV was observed at both temperatures.

**Fig. 5:**
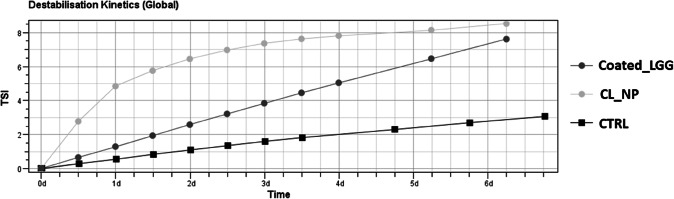
Destabilization kinetics expressed by Turbiscan Stability Index (TSI) of orange fruit juice (CTRL), empty cluster of nanoparticles (CL_NP), and coated *Lacticaseibacillus rhamnosus* GG (Coated_LGG) stored 7 days at 25°C

**Fig. 6:**
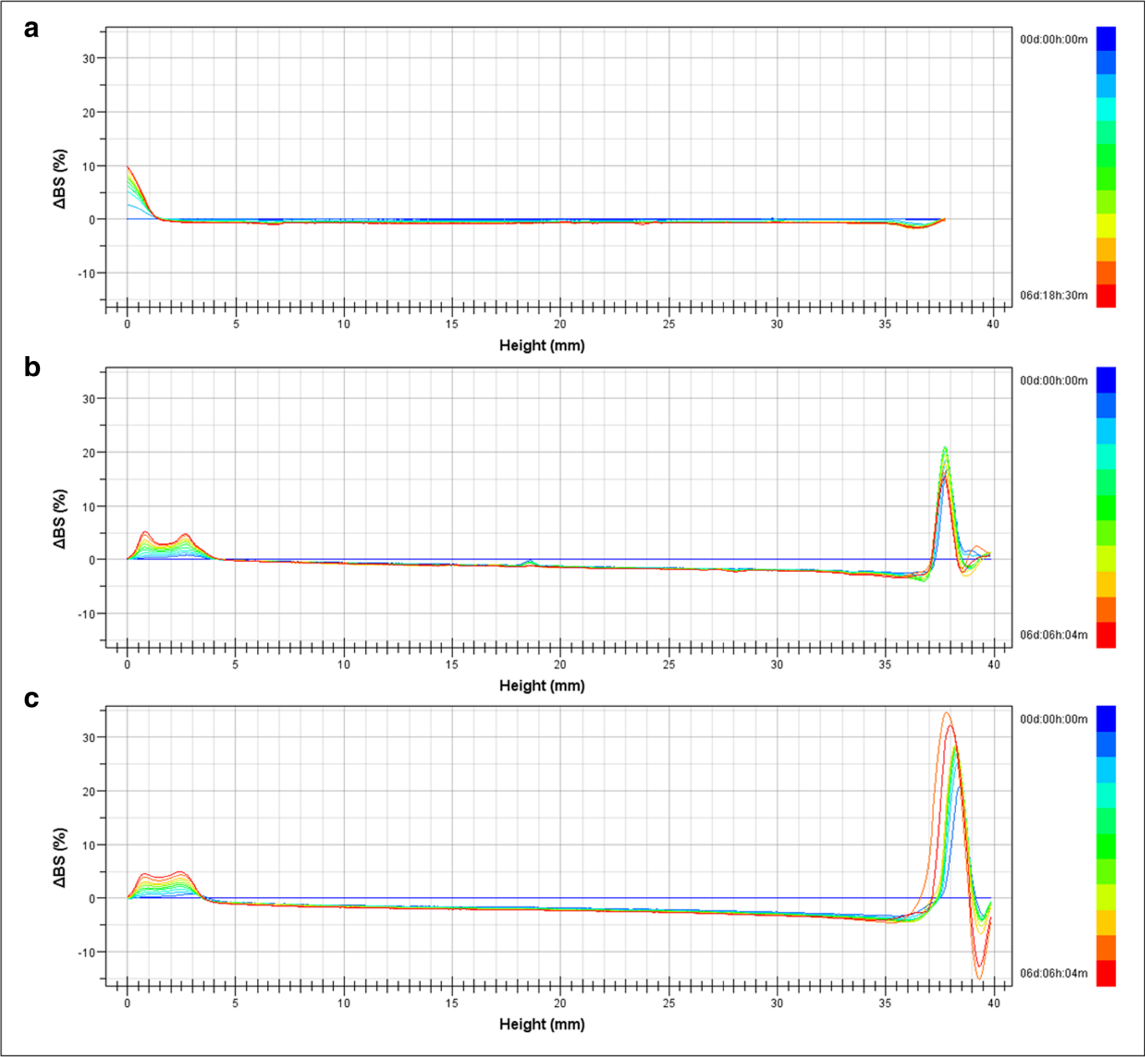
Backscattering profiles (∆BS) of orange fruit juice (CTRL) (**a**), empty cluster of nanoparticles (CL_NP) (**b**), and coated *Lacticaseibacillus rhamnosus* GG (Coated_LGG) (**c**) stored in Turbiscan® at 25°C for 1 week

In addition, CL_NP and Coated_LGG were furtherly analyzed through Turbiscan®, to obtain information on physical stability of colloidal suspensions in terms of particle migration or aggregation (36,37). Turbiscan® Stability Index (TSI) considers all phenomena occurring inside the glass cell at a specific sampling point (37)*.* As shown in the destabilization kinetics, reported in Fig. 5, both CL_NP and Coated_LGG showed higher values compared to fruit juice alone at 25°C, and variation in the TSI was observed after 7 days of storage. The alginate system accelerated the sedimentation of the pulp presented in fruit juice. In order to further investigate the instability phenomena, we also evaluated the BS profiles of the formulations (Fig. 6).

Insignificant instability phenomena (∆BS >10%) was registered for both CL_NP and Coated_LGG (Fig. 6 b and c, respectively) compared to the fruit juice (Fig. 6a), due to reversible particle migration at both the bottom and the top of the cuvette. ∆BS along the glass cell provides information related to the sample homogeneity, confirming the PDI variation observed through PCS. Furthermore, a linear profile for CL_NP and Coated_LGG in the middle of the glass cell was observed, clearly revealing the absence of particle size increase related to aggregation phenomena.

### Counting of Encapsulated LGG and Microbiological Analyses of Juice Samples

The initial bacterial population subjected to encapsulation was around 9.11 log_10_ CFU/mL. High cell entrapping (8.07 log_10_ CFU/g Coated_LGG) was achieved in Coated_LGG, with no significant loss of viability and with about 87% of successfully entrapped cells (data not shown).

**Fig. 7:**
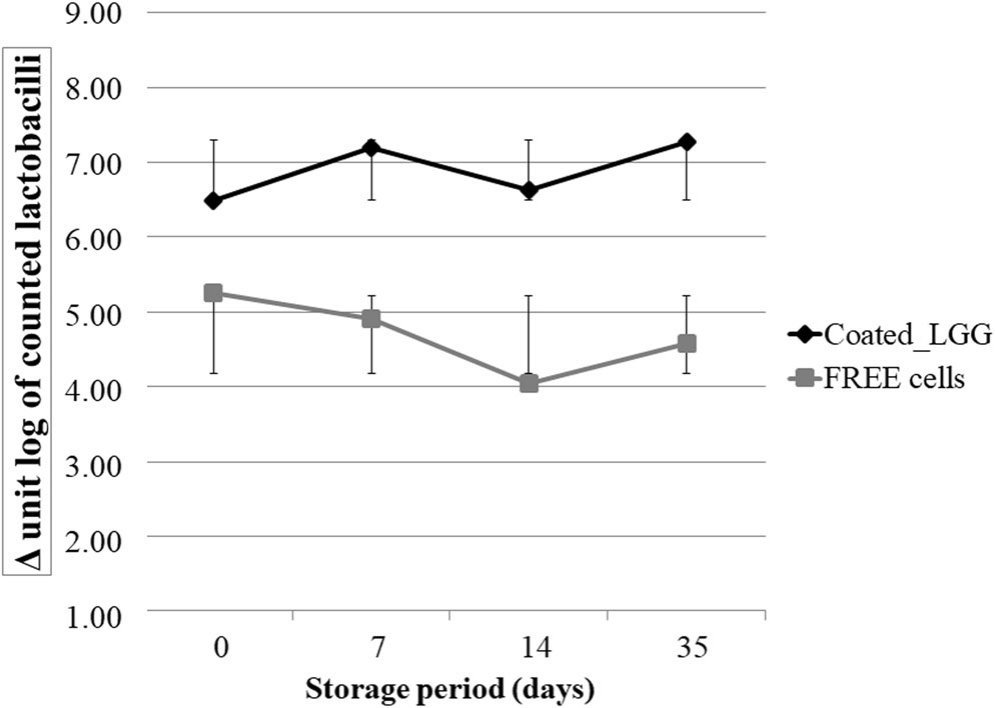
Viability of LGG cells, in free and microencapsulated forms, during refrigerated (5°C) storage (days) in blood orange juices. Data are expressed as differences between lactobacilli densities detected in experimental and control juice samples (uninoculated juice)

The viability of microencapsulated cells was monitored into orange juice during refrigerated storage and compared to viability of free cells added and naturally present in juice, including failed encapsulated cells. Juice samples with no added probiotic bacteria were also analyzed under the same conditions as control. The microbial population dynamics detected in orange juice samples during 35 days of storage at 5°C are shown in Table V, while the survival of LGG, in both experimental samples (with Coated_LGG or free LGG) reported as differences (∆ log) between completely coated cells or free added cells and lactobacilli naturally present (including not coated LGG), is reported in Fig. 7.

**Table V:** Microbial Groups (Expressed as log CFU/mL) in Orange Juice Samples During Storage (at 0, 7, 17, and 35 days) at 5°C

Microbial group	*t*_0_			*t*_7_			*t*_14_			*t*_35_		
	CTRL	Coated_LGG	LGG	CTRL	Coated_LGG	LGG	CTRL	Coated_LGG	LGG	CTRL	Coated_LGG	LGG
Lactobacilli	2.67±0.19^g^	9.15±0.04^a^	7.93±0.05^d^	2.03±0.05^h^	9.22±0.08^a^	6.94±0.13^c^	2.00±0.00^h^	8.63±0.09^b^	6.04±0.04^e^	1.00±0.00^i^	8.28±0.12^bc^	5.58±0.38^f^
*Leuconostoc* spp.	2.81±0.03^d^	2.01±0.59^c^	2.33±0.03^d^	2.10±0.09^d^	2.02±0.15^d^	5.80±0.96^b^	2.07±0.09^d^	2.15±0.07^d^	6.55±0.07^b^	2.00±0.00^d^	2.50±0.02^d^	8.03±0.07^a^
Mesophilic bacteria	1.97±0.58^efg^	2.05±0.29^c^	1.50±0.46^fg^	1.72±0.02^efg^	2.97±0.42^d^	4.16±0.15^c^	2.09±0.09^efg^	2.57±0.29^de^	6.10±0.09^b^	1.31±0.02^def^	2.30±0.35^def^	8.10±0.17^a^
Molds/yeasts	2.70±0.01^c^	3.10±0.09^bc^	3.00±0.25^c^	1.63±0.03^d^	2.09±0.18^cd^	4.06±0.06^ab^	1.50±0.02^d^	4.97±1.16^a^	4.06±0.02^ab^	1.31±0.02^d^	4.79±0.20^a^	4.68±0.14^a^
Psychotropic bacteria	<1^i^	<1^i^	<1^i^	1.03±0.02^h^	2.63±0.02^c^	2.32±0.02^d^	1.32±0.02^g^	3.10±0.09^b^	3.51±0.03^a^	1.49±0.01^f^	1.51±0.03^f^	1.65±0.04^e^

*Means ± standard deviation (SD). Means with different letters within the same row indicate significant difference at p < 0.05*

*CTRL, control; Coated_LGG, coated Lacticaseibacillus rhamnosus GG; LGG, Lacticaseibacillus rhamnosus GG*

The densities of lactobacilli in juice samples, added with Coated_LGG, ranged between about 9.0, at initial time, and 8.3 log CFU/mL after 35 days of refrigerated storage (Table V). During storage, significant differences in lactobacilli densities were observed only at the 14th and 35th days of storage, highlighting a slight decrease, reaching an average value of about 8.6 and 8.3 log CFU/mL, respectively. Our results confirmed previous reports on the effects of microencapsulation on maintenance cell viability into fruit juice for 2 weeks (38). This finding has been ascribed to a more favorable anaerobic microenvironment for bacteria or to a physical barrier against the acidic conditions of fruit juices (39). It is interesting to highlight that the cell density observed after 35 days was almost equivalent to the initial value of added LGG cells, thus revealing that the microcapsules remained intact in the juice and confirming the ability of sodium alginate to protect cells. Juice added with free LGG showed a lower survival rate (Table V), with a constant lactobacillus decrease, from the initial 7.93 CFU/mL to 5.58 CFU/mL at the 35th day of storage. Although several studies reported a good stability of LGG strain in orange juice (40), many other reports highlighted a considerable variability mostly referred to intrinsic parameters of juice (namely microbial loads) or processing and storage temperature (40,41).

Interestingly, our results demonstrated only an insignificant decrease in viability after 7 days of storage; this result was in contrast to literature findings that showed how the viability of free probiotic cultures in fruit juices is difficult to maintain for low pH, high concentration of dissolved oxygen, and low concentrations of free amino acids and peptides (3).

Additionally, in order to obtain a microbiological picture of juice with and without alginate microcapsules, further microbiological analyses were performed. Overall, the microbiological results (Table V) confirmed that all tested orange juices were in compliance with the limits dictated by the CE Regulation 2073/05 (41). In control samples (CTRL), *Leuconostoc* spp. counts showed a slight decrease after 7 days of storage to remain almost constant up to end of storage (Table V). Mesophilic counts showed a swinging trend with an initial slight decrease, an increase up to the 14th day of storage, and further decrease up to the 35th day of storage when the value of 1.31 log CFU/mL was reached. An initial reduction (1.07 log unit) was observed for mold/yeast densities, which reached the value of 1.30 log CFU/mL after 35 days of storage (Table V). Psychotropic bacteria, not detectable at initial time, reached the final values of 1.50 log CFU/mL. Zooming on inoculated juices *Leuconostoc* showed a stable density, of about 2 log CFU/mL until the end of the storage, although microbiological count was somehow higher for the Coated_LGG juices compared to the CTRL juice. Regarding mesophilic bacteria, an increase of about 1 log unit was observed after 7 days, followed by a decrease after 14 and 35 days (Table V). Fluctuations in the counts of molds and yeasts were registered with a peak density recorded (about 5.00 log CFU/mL) after 14 days and a further decrease (4.80 log CFU/mL) at the end of storage. The psychrotrophic bacteria reached a value of 3.10 log CFU/mL after 14 days of storage and a subsequent decrease (1.51 log CFU/mL) after 35 days.

The addition of free LGG cells into orange juice contributed to an increase of other microbial groups. In details, a constant increase was observed for *Leuconostoc* spp., along the storage period, reaching the value of 8.00 log CFU/mL after 35 days (Table V). Regarding mesophilic bacteria, about 2 log unit increase at any sampling time was observed, reaching, after 35 days, a value of 8.00 log CFU/mL, while a lower increase (1.06 log units) was detected for mold/yeast counts that reached the value of 4.68 log CFU/mL at the end of storage (Table V). After 7 days, the psychrotrophic bacteria increased to reach the highest density (3.51 log CFU/mL) after 14 days and a final value of 1.65 log CFU/mL (Table V).

Overall, the results of the present study highlighted that the microencapsulation represents a promising approach for juice preservation, to improve the stability of the final product. Although fruit juice is a suitable matrix for spoilage microbial growth and supporting the growth of molds/yeasts, heterofermentative LAB species, and above all *Leuconostoc* spp. (42,43), the microencapsulation strategy was found more effective for reducing the overgrowth of microorganisms, compared to the addition of free LGG. Even if the yeasts’ presence may cause color loss and the formation of a sediment at the bottom, with consequent turbidity, flocculation, pellicles, and clumping, in our study, no visual differences among tested juice samples were revealed (Supplementary Figure 2).

### pH and Total Soluble Solid (°Brix)

The initial pH values of blood orange juice samples were between 2.75 and 3.45. Overall, the pH value remained almost constant in treated and non-treated juices at any sampling time, in agreement with previous studies. Champagne and Gardner (44) studied the stability at 4°C of nine probiotic lactobacilli in a drink, composed of 10 fruits and dairy ingredients, observing that the pH of samples remained unchanged after 28 days of storage due to the weak metabolic activity of the lactobacilli at 4°C. Similarly, the same authors found a variation in pH between 3.63 and 3.90, when *L. rhamnosus* R0011 was inoculated (at 4.5×10^9^ CFU/250 mL) into an apple-pear-raspberry juice blend (45). The inoculated juices showed pH values less than 0.1 unit lower than the non-inoculated juices. In the same way, the total soluble solids did not significantly change in any tested samples during storage.

## CONCLUSION

This study was aimed at the preparation of alginate-based system for probiotic delivery into juices. The BBD revealed that polymer and surfactant concentrations are the main factors determining particle properties. The optimized particles were prepared using 2% alginate and 0.4% Span 80 which led to particles with a diameter ~1000 nm. The experimental data of the optimized formulation were in accordance with the predicted ones, suggesting that the experimental design was a valid approach for the investigation and optimization of new formulations. Additionally, the morphological analysis revealed the capsular structure of the unloaded systems, showing a cluster organization in aqueous media. The optimized formulation loaded with the probiotic (Coated_LGG) was found to be homogeneous with a slight increase in mean size in comparison to unloaded CL_NP. Indeed, the elongated elliptical shape revealed that the coating process is one to one for bacterial cells.

Microbiological evaluation revealed that the encapsulation assured the survival of Coated_LGG, in a 100% orange juice for 28 days. Moreover, results indicated that the microencapsulated bacteria preserved the macroscopic properties and the microbiological characteristic of orange juice and assured the required amount of probiotic live cells to obtain a temporary colonization of the intestine. Further studies are required to test the survival of microencapsulated strain through the gastrointestinal tract in vitro.

## Supplementary information

Supplementary Figure 1. Correlation between predicted and actual values for particles size obtained combining Alginate, Span80 and A/O ratio at their three different levels. (PNG 43 kb)

High resolution image (TIF 190 kb)

Supplementary Figure 2. Macroscopic observation of the orange fruit juice (CTRL); free LGG strain inoculated in orange fruit juice (LGG); Coated-LGG inoculated in orange fruit juice (C-LGG) at a) 0 time; and after 14 b) and 35 c) days of storage at refrigerated temperature. (PNG 412 kb)

High resolution image (TIF 574 kb)

ESM 1 (DOCX 24.8 kb)

## Abbreviations

BBD: Box-Behnken Design
BS: Backscattering
CFU: Colony-forming unit
CL_NP: Cluster of nanoparticles
Coated_LGG: Coated *Lacticaseibacillus rhamnosus* GG
CPPs: Critical process parameters
CQAs: Critical quality attributes
CTRL: Control
DLS: Dynamic light diffusion
DoE: Design of the experiment
EDL: Electric double layer
LAB: Lactic acid bacteria
LGG: *Lacticaseibacillus rhamnosus* GG
MCs: Microcapsules
NPs: Nanoparticles
PCS: Photon correlation spectroscopy
PDI: Polydispersity index
QTTP: Quality target profile
SD: Standard deviation
T: Transmission
TEM: Transmission electron microscopy
TSI: Turbiscan Stability Index
TSS: Total soluble solids
ZP: Zeta potential
