# Supplementary figures and images for: Coating Lacticaseibacillus rhamnosus GG in Alginate Systems: an Emerging Strategy Towards Improved Viability in Orange Juice
Source: AAPS PharmSciTech. 2021 Apr 5;22(3):123. doi: 10.1208/s12249-021-01996-x (PMC8021512; doi:10.1208/s12249-021-01996-x)

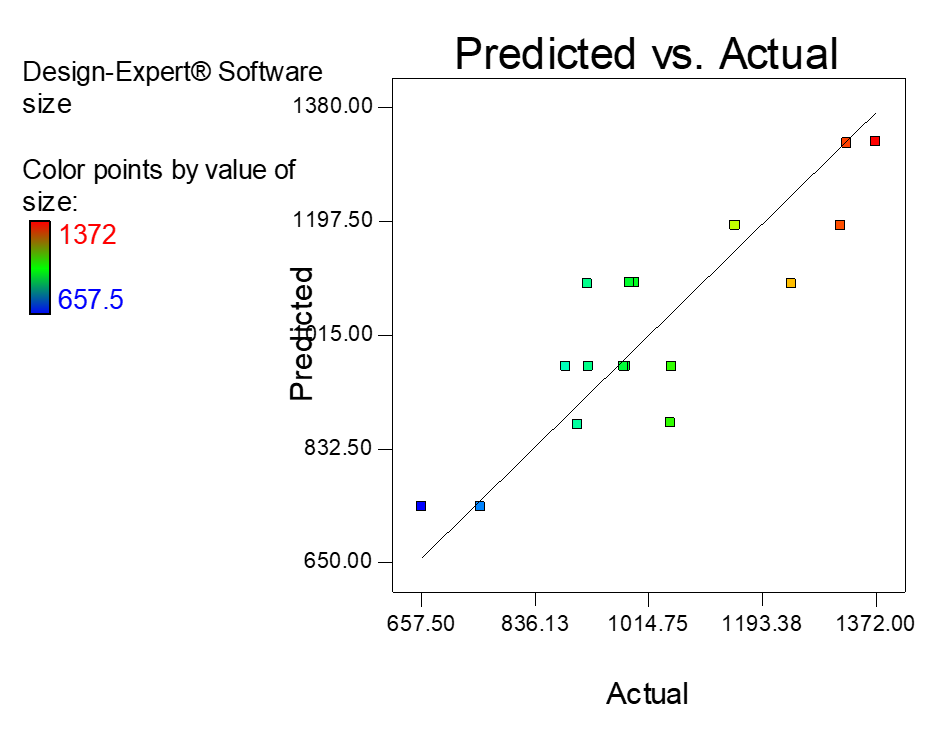

Supplement: Supplementary file 1 — Correlation between predicted and actual values for particles size obtained combining Alginate, Span80 and A/O ratio at their three different levels. (PNG 43 kb) [file 12249_2021_1996_Fig8_ESM.png]

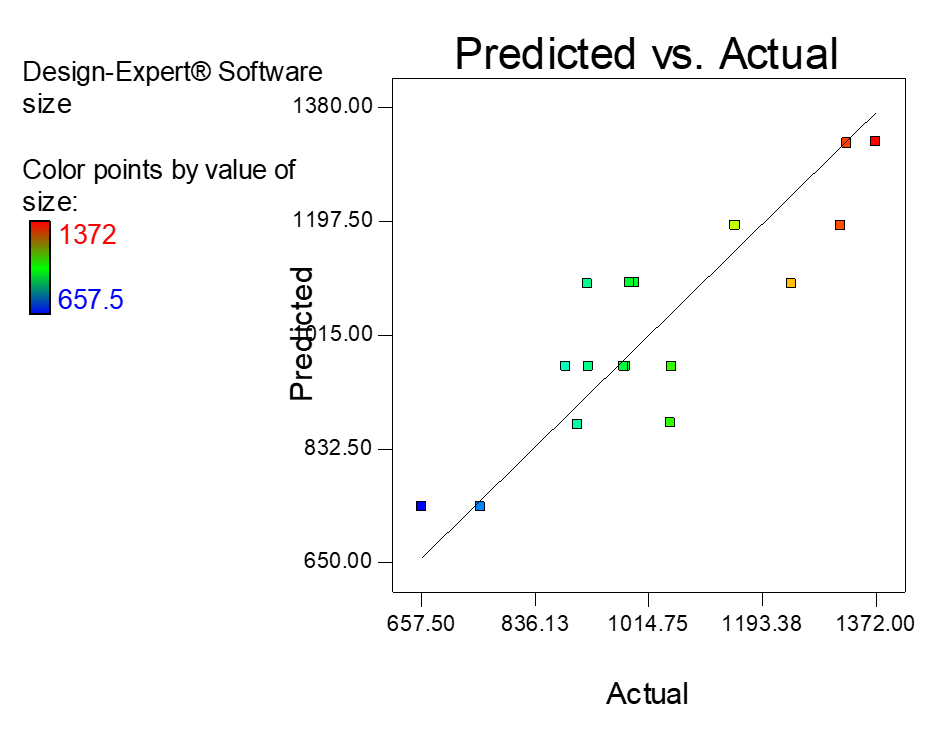

Supplement: Supplementary file 2 — High resolution image (TIF 190 kb) [file 12249_2021_1996_MOESM1_ESM.tif]

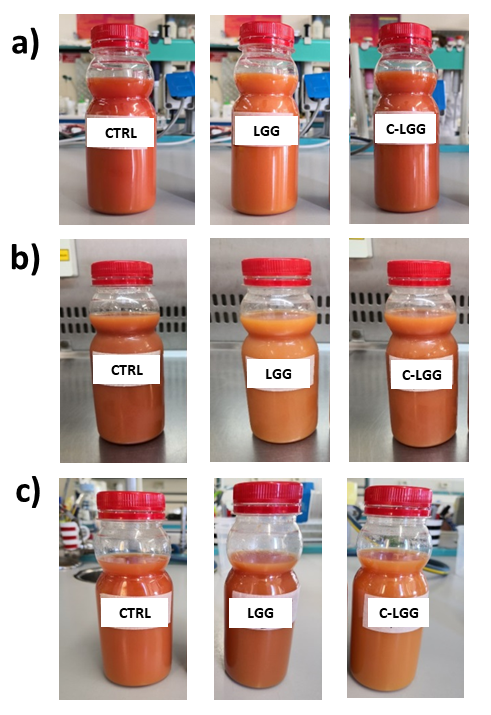

Supplement: Supplementary file 3 — Macroscopic observation of the orange fruit juice (CTRL); free LGG strain inoculated in orange fruit juice (LGG); Coated-LGG inoculated in orange fruit juice (C-LGG) at a) 0 time; and after 14 b) and 35 c) days of storage at refrigerated temperature. (PNG 412 kb) [file 12249_2021_1996_Fig9_ESM.png]

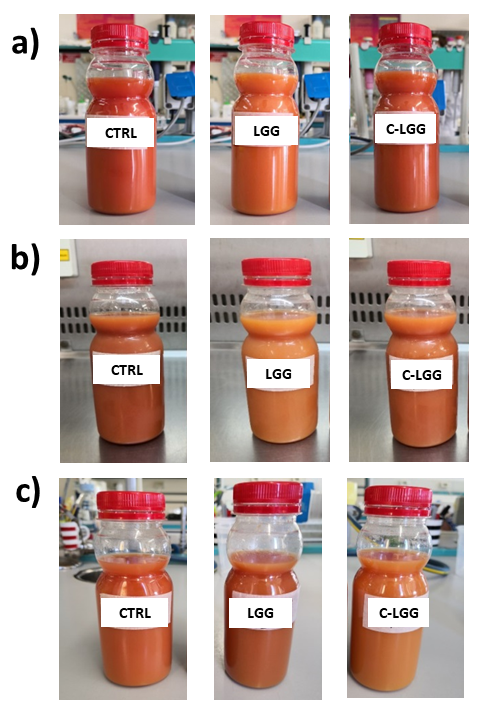

Supplement: Supplementary file 4 — High resolution image (TIF 574 kb) [file 12249_2021_1996_MOESM2_ESM.tif]
