# Supplementary material for: Coating Lacticaseibacillus rhamnosus GG in Alginate Systems: an Emerging Strategy Towards Improved Viability in Orange Juice
Source: AAPS PharmSciTech. 2021 Apr 5;22(3):123. doi: 10.1208/s12249-021-01996-x (PMC8021512; doi:10.1208/s12249-021-01996-x)
Supplement: Supplementary file 5 — (DOCX 24.8 kb) [file 12249_2021_1996_MOESM3_ESM.docx]

**Supplementary Table I.** Coded levels of independent variables and response variables.

| **Independent Variables^a^** | **Goals** | **Lower limit** | **Upper limit** |
| --- | --- | --- | --- |
| **X_1_** | MAXIMIZE | 0.1 | 0.5 |
| **X_2_** | MAXIMIZE | 0.5 | 2 |
| **X_3_** | IN RANGE | 1:20 | 1:6 |
| **Dependent Variables^b^** |  |  | |
|  |  |  |  |
|  |  |  |  |
| **Y_1_** | TARGET=1000 | 657.5 | 1372 |
| **Y_2_** | MINIMIZE | 0.149 | 1 |

*^a^Independent variables: X_1_= [Span 80]; X_2_ = [Alginate]; X_3_= Water: Oil*

*^b^Dependent Variables: Y_1_= Size(nm); Y_y_= Pdi;*

**Supplementary Table II.** Process parameters investigated to tune the ionotropic gelation technique.

| **Magnetic Stirring** | **rpm**  100 – 18.000 | |
| --- | --- | --- |
| **Dripping distance** | **cm** | |
|  | 5-50 | |
| **Purification by centrifuge** | **rpm** | **Time (min)** |
|  | 1.000-10.000 | 5-30 |
| **Crosslinking Time** | **time (min)** | |
|  | 15-60 | |
